# Referential focus moderates depression-linked attentional avoidance of positive information

**DOI:** 10.1016/j.brat.2017.03.004

**Published:** 2017-06

**Authors:** Julie Lin Ji, Ben Grafton, Colin MacLeod

**Affiliations:** a Medical Research Council Cognition & Brain Sciences Unit, University of Cambridge, United Kingdom; b Centre for the Advancement of Research on Emotion, School of Psychology, University of Western Australia, Australia; c School of Psychology, Babes-Bolyai University, Romania

**Keywords:** Depression, Attentional bias, Self-referential processing, Dot-probe task

## Abstract

While there is consensus that depression is associated with a memory bias characterized by reduced retrieval of positive information that is restricted to information that had been self-referentially processed, there is less agreement concerning whether depression is characterized by an attention bias involving reduced attention to positive information. However, unlike memory research, previous attention research has not systematically examined the potential role of referential processing focus. The present study tested the hypothesis that evidence of depression-linked attentional avoidance of positive information would be more readily obtained following the self-referential processing of such information. We assessed attentional responding to positive information (and also to negative information) using a dot-probe procedure, after this information had been processed either in a self-referential or other-referential manner. The findings lend support to the hypothesis under scrutiny. Participants scoring high in depression score exhibited reduced attention to positive information compared to those scoring low in depression score, but only when this information had been processed in a self-referential manner. These findings may shed light on the mechanisms that underpin attentional selectivity in depression, while potentially also helping to account for inconsistencies in previous literature.

## Highlights

• First systematic investigation of the role of referential processing focus in moderating depression-linked attentional bias.
• Reduced attention to self-referential positive information was exhibited by high relative to low depression individuals.
• No evidence of a depression-linked reduction in attention to other-referential positive information was found.
• Findings shed light on the mechanisms that underpin attentional selectivity in depression.
• Findings also help to account for inconsistencies in previous literature.

Cognitive theories of depression posit that systematic biases in the processing of emotional information distort subjective reality in ways that elevate negative emotional disposition (Beck, 1976, Ingram, 1984, Teasdale, 1988). For example, Beck argues that depressed individuals possess schemas that guide the operation of memory and attention in ways that result in processing advantages for schema congruent information (Beck, 1976). Considerable research effort over the past three decades has sought to determine whether biases in attention and memory are characteristic of elevated vulnerability to depression (Mathews and MacLeod, 1994, Mathews and MacLeod, 2005). While clear experimental evidence of a depression-linked memory bias has been obtained, there has been debate concerning whether or not depression is characterized by an attentional bias (Mineka et al., 1998, Williams et al., 1997).

Depression-linked memory bias is more evident for emotionally positive information than for emotionally negative information. Specifically, relatively impaired memory for positive information (rather than relative enhanced memory for negative information) has been observed in individuals with clinical levels of depression compared to healthy controls (Breslow et al., 1981, Burt et al., 1995, Dozois and Dobson, 2001, McDowall, 1984), as well as individuals experiencing depressed mood (dysphoria) compared to healthy controls (Finkel et al., 1982, Ingram et al., 1983, Murray et al., 1999). This pattern of bias has also been observed in individuals with experimentally induced negative mood compared to those with experimentally induced positive mood (Isen, Shalker, Clark, & Karp, 1978; Study 2; Nasby & Yando, 1982; Study 1).

Although there is general agreement that depression is characterized by biased memory processing (c.f. Matt et al., 1992, Mathews and MacLeod, 2005), it is important to note that such effects are only reliably observed under a very particular processing condition (Blaney, 1986, Matt et al., 1992). Critically, several reviews have noted that depression-linked memory selectivity is robustly observed only after emotional information has initially been processed in a self-referential manner (Blaney, 1986, Gaddy and Ingram, 2014, Matt et al., 1992). In a typical experimental paradigm, referential processing focus is manipulated by having participants rate a list of positive and negative personality attribute words according to whether or not each attribute is descriptive of the self (self-referential condition) or descriptive of a familiar other (other-referential condition). Participants’ memory for the stimuli is subsequently tested using explicit recall paradigms. In studies where referential focus has been left unconstrained during encoding, evidence of depression-linked memory bias has been mixed. While some studies do find memory impairment for positive information in clinically depressed individuals compared to non-depressed controls (Breslow et al., 1981), others fail to observe any memory differences between depressed and non-depressed controls (Hasher, Rose, Zacks, Sanft, & Doren, 1985). However, when investigators have experimentally elicited self-referential processing of the stimuli prior to the memory test, a depression-linked bias reflecting reduced memory for positive information has been observed. No such effect is evidenced when the stimuli initially has been processed in an other-referential manner (Bradley and Mathews, 1983, Kuiper and Derry, 1982). Such findings have led investigators to conclude that this memory bias is driven by a distorted self-schema in depressed individuals that is characterized by a lack of positive information about the self (Dozois & Dobson, 2001).

Evidence of this depression-linked memory bias has generally been consistent across studies (Mathews & MacLeod, 2005). In contrast, while meta-analytic investigations have revealed that across studies, on average, there is evidence of a depression-linked attentional bias (Peckham et al., 2010, Winer and Salem, 2016), whether or not such attentional bias is observed in an individual study has been highly variable (Gotlib and Joormann, 2010, Mathews and MacLeod, 2005). Some studies using variants of the attention probe task (MacLeod, Mathews, & Tata, 1986) to assess attention bias report findings that individuals experiencing depressed mood (Bradley et al., 1998, McCabe and Toman, 2000), or clinical levels of depression (Gotlib, McLachlan, & Katz, 1988), exhibit reduced attention to positive information compared to healthy controls. However, many more studies using similar attentional assessment approaches have failed to find any depression-linked individual differences in the attentional processing of positive (or negative) information (Mogg et al., 1995, Bradley et al., 1997; for a review see; Mogg & Bradley, 2005).

Researchers have speculated about the precise processing conditions under which depression-linked attentional bias may be observed. The idea that has received most experimental scrutiny is that depression-linked attentional bias may be more evident at longer stimulus exposure durations (e.g. 1000 ms) than shorter stimulus exposure durations (e.g. 500 ms; Mogg & Bradley, 1998), perhaps reflecting increased attentional disengagement from positive information, rather than reduced attentional engagement with such positive emotional information (Sanchez et al., 2013, Koster et al., 2004). Consistent with this idea, the majority of attentional probe studies reporting evidence of depression-linked attentional bias have employed stimulus exposure durations of 1000 ms or above (c.f. Gotlib and Joormann, 2010, Mogg and Bradley, 2005), while failures to demonstrate this effect have employed stimulus exposure durations of 500 ms or shorter (Donaldson, Lam, & Mathews, 2007). However, this hypothesis cannot fully account for the observed inconsistencies, given that several studies employing stimulus exposure durations of 1000 ms or more have failed to obtain evidence of a depression-linked attention bias (Mogg et al., 2000, Neshat-Doost et al., 2000), and on occasions, evidence of depression-linked attentional bias has been obtained using 500 ms stimulus exposure durations (Mathews et al., 1996, Shane and Peterson, 2007). Hence, cross-study variation in the use of shorter and longer stimulus exposure durations cannot suffice to explain the observed inconsistency concerning depression-linked bias in attentional responding to emotional information.

Intriguingly, despite evidence that depression-linked memory impairment for positive information is restricted to conditions in which information has been processed in a self-referential manner (Blaney, 1986, Gaddy and Ingram, 2014, Matt et al., 1992), no research has tested the possibility that evidence for a depression-linked reduction in attention to positive information may be more readily obtained following self-referential processing of such information. Indeed, if depression-linked information processing bias is driven by the operation of a depressogenic self-schema that lacks positive information, as previous theorists have argued, then reduced attention to positive information in high-depression compared to low-depression participants would be disproportionately evident when this self-schema has been activated by having participants process this emotional information in a self-referential rather than other-referential manner. Importantly, the degree to which participants have engaged in the self-referential processing of stimulus information is likely to have varied in an uncontrolled manner across prior experiments assessing depression-linked attentional bias, because referential processing focus had not been systematically constrained or manipulated. Such variability may have contributed to the pattern of inconsistent findings observed within this literature to date.

The present study was designed to directly test this referential processing focus hypothesis. Using the well-established dot-probe task, we contrasted patterns of attentional bias to emotional information in participants who scored either high or low on the 21-item Depression subscale of the Depression, Anxiety & Stress Scale (DASS-21; Lovibond & Lovibond, 1995). To dissociate selective attentional responding to positive information from selective attentional responding to negative information, it is necessary to pair each type of emotional stimulus with an unemotional stimulus. While some researchers carrying out such attentional assessment have employed neutral words as the unemotional stimulus, others have expressed concern that words intended to be neutral may, for some participants, have emotional significance due to past associations and experience (Grafton, Southworth, Watkins, & MacLeod, 2016), particularly in depressed individuals who are known to display negatively biased interpretations of neutral information (Lawson & MacLeod, 1999). Thus, to eliminate this possibility, these investigators have used meaningless non-words as the non-emotional stimulus, and have successfully dissociated biased attentional processing of positive information from biased attentional processing of negative information (e.g. Grafton et al., 2012, Grafton et al., 2016). Hence, we adopted this approach in the current dot-probe task, by presenting emotionally positive and negative words that each were paired with a non-word letter string, for 500 ms or 1000 ms, and inferring the resulting attentional distribution between members of the stimulus pair from relative discrimination latencies for probes that then appeared in the locus of either stimulus. Of critical importance, to determine whether depression-linked attentional bias is more evident when stimuli have initially been processed in a self-referential rather than an other-referential manner, this attentional bias assessment was carried out *after* participants had first judged whether each stimulus word was descriptive of the self (self-referential condition) or was descriptive of another specified individual (other-referential condition). In keeping with the approach adopted in previous research, the specified individual used in the other-referential condition was a familiar public figure (Kuiper and Derry, 1982, Pyszczynski et al., 1989). The hypothesis under test predicts that evidence of reduced attention to positive information in individuals with high levels of depression compared to those with low levels of depression would be disproportionately great under the self-referential processing condition than under the other-referent processing condition.

## 1 Method

### 1.1 Participants

Participants were forty-five introductory psychology students at the University of Western Australia. We sought to distinguish participants who scored relatively high and relatively low on the depression subscale of the 21-item Depression, Anxiety, and Stress Scale (DASS-21; Lovibond & Lovibond, 1995). Thus, we screened 700 first year psychology students on this instrument. Anticipating regression to the mean between the time of this screening and the test session (scheduled approximately 3–5 weeks later), only those individuals who scored either in the top or bottom 20% of the resulting screening score distribution were invited to participate in the study. This resulted in the recruitment of 23 participants with high DASS-21 depression scores at screening (score range = 14–42, *M* = 25.65, *SD* = 7.45), and 22 with low DASS-21 depression scores at screening (score range = 0–4, M = 1.81, *SD* = 1.22), giving rise to the between group factor Depression Level (High depression vs. Low depression). Participants from each group were randomly allocated to either the self-referential processing condition or to the other-referential processing condition, giving rise to a second factor, nested within the Depression Group factor, which is here termed Processing Condition (Self-referential processing vs. Other-referential processing).

As intended, DASS depression scores differed as a function of the Depression Level factor, *F* (1, 44) = 75.38, *p* < 0.001, *η*^*2*^_*p*_ = 0.65, but not as a function of the Processing Condition factor, *F* (1, 44) = 0.005, *p* = 0.94, or as an interactive function of both factors, *F* (1, 44) = 0.02, *p* = 0.89. An equivalent analysis carried out on age provided the reassurance that participant age did not differ as a function of either factor or their interaction (in all cases *p* > 0.05). Gender ratio did not differ significantly across the four cells, resulting from the nested combination of these two factors, all *χ*^2^ (1, 45) < 0.08, *p* > 0.78.

### 1.2 Materials

#### 1.2.1 Experimental stimuli

We required 96 pairs of letter strings in which one member was a trait descriptive word of either negative or positive emotional valence, while the other was a length-matched non-word, which was devoid of emotional content. We selected 48 positive and 48 negative trait descriptor words, based on the “Likeableness” norms for 555 personality-trait words provided by Anderson (1968). Trait “likeableness” in Anderson (1968) was indexed by ratings given to each word on a 6-point Likert scale, ranging from 1 (least likeable) to 6 (most likeable). Our 48 positive and negative trait word members differed significantly in mean Likeableness rating, *t* (94) = 33.31, *p* < 0.001. The mean Likeableness scores for our positive trait words was *M* = 4.56, *SD* = 0.44, and for our negative words was *M* = 1.51, *SD* = 0.45. Each of these words was paired with a length-matched non-word, created by randomly selecting characters from the alphabet without replacement, thereby giving rise to the 96 letter string pairs. The full set of experimental word stimuli is provided in the Appendix.

#### 1.2.2 Depression, anxiety, and stress scale

Participants’ levels of depression symptoms were assessed using the Depression subscale of the 21-item Depression, Anxiety, and Stress Scale (DASS-21; Lovibond & Lovibond, 1995). This sub-scale requires participants to respond to seven statements describing various depressive symptoms on a four-point scale, ranging from 0 (Does not apply to me at all) to 3 (Applies to me very much). The DASS-21 has been shown to have both good reliability and validity (Henry and Crawford, 2005, Osman et al., 2012).

#### 1.2.3 Experimental hardware

The attentional probe task was run using a Hewlett-Packard PC, and presented on a 22-inch widescreen color monitor set at a resolution of 1680 × 1050 pixels. All responses were made using a standard two-button mouse.

#### 1.2.4 Experimental task

To ensure that participants initially processed the stimulus words in either a self-referential or other-referential manner, a block of referential judgment trials preceded each block of 96 probe trials. These referential judgment trials required participants either to judge whether each word described themselves, or a familiar television news anchor, depending on whether the participant had been assigned to the Self-referential or Other-referential processing condition, respectively. Across the full task, six blocks of attentional probe trials, each preceded by a block of referential judgment trials, were delivered. The specific nature of each trial type was as follows.

##### 1.2.4.1 Referential judgment trials

For participants assigned to the Self-referential processing condition, each block of referential judgment trials commenced with the onscreen instruction “*Now you will be asked to judge whether words describe you. Press any key to continue*”. Each referential judgment trial then presented a single word from the stimulus set in the center of the screen, where it remained until participants indicated whether this word described them. The next word then immediately appeared for rating. Responses were made using the right and left mouse buttons, to respectively indicate a “yes” response (word is self-descriptive) and a “no” response (word is not self-descriptive). Participants assigned to the Other-referential processing condition were required at the beginning of the test session to select, from a list of Western Australian television news anchors, one individual they were familiar with but felt emotionally neutral towards. For these participants, each block of referential judgment trials began with the onscreen instruction “*Now you will be asked to judge whether the words describe television news anchor xxx. Press any key to continue*”, where xxx was the name of the television news anchor the participant had selected. The referential judgment trials for participants in this Other-referential processing condition presented single words, just as in the Self-referential processing condition, but participants now were required to indicate whether or not each word was descriptive of the target television news anchor. Again, responses were made using the right and left mouse buttons, to respectively indicate a “yes” response (word describes television news anchor) and a “no” response (word does not describe television news anchor).

##### 1.2.4.2 Attentional probe trials

In the attention probe task, participants were first presented with a pair of emotionally discrepant stimuli, and were then required to discriminate the identity of a small visual probe that appeared in the locus of either preceding stimuli. It is assumed that probe discrimination latencies will be relatively speeded for probes that appear in the region where attention is focused prior to probe onset. Hence, relative discrimination latencies for probes that appear in the locus of differentially valenced members of these stimulus pairs served to reveal how attention was distributed between them, and thereby index selective attentional responding to emotional information.

Each block of attentional probe trials commenced with the onscreen instruction “*Now you will complete a set of dot-probe trials. Press any key to continue*”. On each trial, a central fixation cross first was displayed for 500 ms, followed by the presentation of one of the letter string pairs, one member appearing just above and the other just below the locus of the previously presented fixation cross. The word member of the pair appeared in the upper or lower position with equal frequency. The vertical distance between the two letter strings was 3 cm, leading to a visual viewing angle of just <3° at the instructed viewing distance of 60 cm. The letter string pair remained on screen for either 500 ms or 1000 ms with equal frequency, before disappearing. A small visual probe then was presented in the locus where either of the preceding letter strings had been shown, and this probe appeared with equal frequency in the locus of the word or the non-word member of this letter string. This probe comprised of either a single small dot, or a pair of immediately adjacent dots, with equal probability. Participants were required to discriminate which of these two probes was presented as quickly and accurately as possible, by pressing the left mouse button to indicate one dot, or the right mouse button to indicate two dots. The latency to make each probe discrimination response was recorded.

### 1.3 Procedure

Participants were tested individually. Upon arrival participants completed the DASS-21 questionnaire to verify that individuals in the High depression and Low depression groups respectively remained in the upper and lower halves of the DASS-21 score distribution observed at test time. Participants were then seated approximately 60 cm viewing distance from the computer screen. Participants in the Self-referential processing condition were told that they would complete two types of tasks, one requiring them to judge whether or not presented words were self-descriptive, and the other requiring them to discriminate the identity of small visual probes presented after pairs of letter strings. Participants in the Other-referential processing condition were first asked to select an individual from a list of Western Australian news anchors that they were familiar with but felt emotionally neutral towards. They then were told that they would complete two types of tasks, one requiring them to judge whether or not presented words were descriptive of this chosen news anchor, and the other requiring them to discriminate the identity of small visual probes presented after pairs of letter strings. All participants were advised that the experiment involved six blocks, each beginning with a set of referential judgment trials, followed by a set of probe discrimination trials. After the referential judgment procedure was explained, participants practiced the dot-probe component of the task over 24 practice trials. Each practice trial consisted of an emotionally neutral word letter string, not used in the actual dot-probe task, paired with an emotionally neutral non-word letter string. The experimental task was then completed, following which participants were debriefed.

### 1.4 Data analysis plan

Probe discrimination latency data can serve to index attentional selectivity only when participants accurately perform the probe discrimination task, and minimum accuracy requirement was set at 90%. Latencies from correct probe discrimination trials were used to compute two measures of attentional selectivity, after first excluding outlying values in accordance with prior conventions.^1^

The first bias index used latencies from trials in which the word member of the letter string pair was positive. This indexed attentional bias to positive words by expressing the degree to which discrimination latencies for probes presented in the locus of these positive words were speeded, relative to probes presented in the locus of the non-word members of these pairs. A higher score on this index signifies more attention to emotionally positive than to unemotional information. The equation can be formally expressed as:

**Attentional Bias to Positive Words Index** = RT for probes in the locus of non-words paired with positive words – RT for probes in locus of positive words.

The second bias index used latencies from trials in which the word member of the letter strings pair was negative. This indexed attentional bias to negative words by expressing the degree to which discrimination latencies for probes presented in the locus of these negative words were speeded, relative to probes presented in the locus of the non-word members of such pairs. A higher score on this index signifies greater attention to emotionally negative than to unemotional information. Formally, the equation can be expressed as:

**Attentional Bias to Negative Words Index** = RT for probes in the locus of non-words paired with negative words – RT for probes in locus of negative words.

## 2 Results

Two participants initially selected on the basis of their high DASS-21 scores at screening obtained DASS-21 scores that fell below the median score at test time, and two participants initially selected on the basis of their low DASS-21 scores at screening obtained DASS-21 scores that fell above the median score at test time. These four participants were consequently excluded from our analyses. In addition, three participants failed to achieve the required accuracy level of 90% on the attentional probe task, thus these participants also were excluded from further consideration. The remaining participants performed the task with a high level of accuracy (*M* = 97.50%, *SD* = 2.29), indicating appropriate compliance with task instructions. Accuracy rates did not differ as a function of Depression Level, *F* (1, 34) = 0.85, *p* = 0.36, Processing Condition, *F* (1, 34) = 0.01, *p* = 0.93, or from an interaction involving these factors *F* (1, 34) = 0.02, *p* = 0.89.

### 2.1 Analysis of attentional bias to negative words index scores

The Attentional Bias to Negative Words Index scores, observed under each condition, are shown in Table 1.

These bias index scores were subjected to a 2 × 2 x 2 mixed design ANOVA that considered the two between-group factors Depression Level (High depression vs. Low depression) and Processing Condition (Self-referential processing vs. Other-referential processing), and the within-group factor Exposure Duration (500 ms vs. 1000 ms). This analysis revealed a two-way interaction between Depression Level and Exposure Duration, *F* (1, 34) = 6.77, *p* = 0.01, *ƞ*^*2*^_*p*_ = 0.17, reflecting relatively greater evidence of attentional vigilance for negative stimuli in the High depression group compared to the Low depression group, when stimuli were exposed for 1000 ms compared to when they were exposed for only 500 ms. Critically, we found no evidence whatsoever that the impact of Depression Level on Attentional Bias to Negative Information Index scores was modified by the Processing Condition factor, either as a two-way interaction, *F* (1, 34) < 0.001, *p* = 0.99, or a three-way interaction involving Exposure Duration, *F* (1, 34) = 0.14, *p* = 0.71. As such, attentional processing of negative information was unaffected by the self-referential processing or other-referential processing of such information prior to attention assessment.

### 2.2 Analysis of attentional bias to positive words index scores

The Attentional Bias to Positive Words Index scores, observed under each condition, are shown in Table 2.

**Fig. 1:**
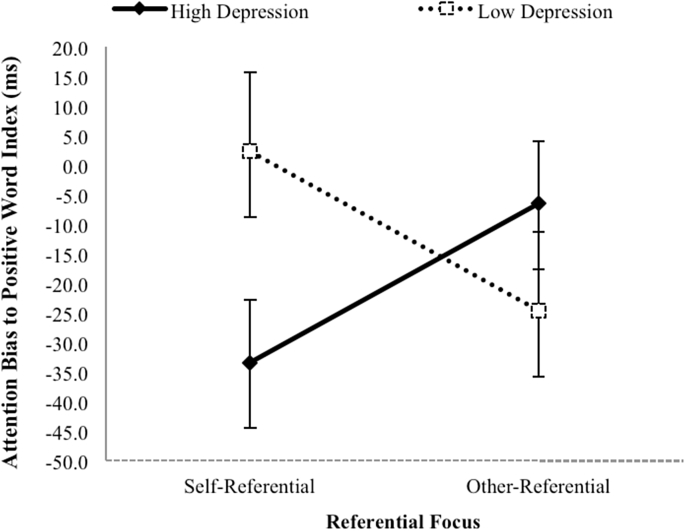
Means and standard errors on the Attention Bias to Positive Words Index.

These bias index scores were subjected to the same 2 × 2 x 2 mixed design ANOVA, again considering the two between-group factors Depression Level (High depression vs. Low depression) and Processing Condition (Self-referential processing vs. Other-referential processing), and the within-group factor Exposure Duration (500 ms vs. 1000 ms). This analysis confirmed the predicted two-way interaction between Depression Level x Processing Condition, *F* (1, 34) = 5.41, *p* = 0.03, *ƞ*^*2*^_*p*_ = 0.14, which was the only effect to achieve significance. This significant two-way interaction is shown in Fig. 1.

As can be seen, the nature of this interaction is consistent with the hypothesis that a depression-linked attentional bias involving a relative reduction in attention to positive stimuli would be disproportionately great in the Self-referential processing condition compared to the Other-referential processing condition. In fact, such a pattern of means was observed only in the Self-referential processing condition, and there was no evidence whatsoever of reduced attention to positive information in the High depression group compared to the Low depression group in the Other-referential processing condition.

## 3 Discussion

The findings of the present study support the hypothesis that, as is the case with depression-linked memory bias, referential-focus during the processing of emotional information moderates the subsequent expression of depression-linked attentional bias to such information. As predicted, evidence was obtained of a depression-linked reduction in attention to positive information, and consistent with the novel hypothesis under test, such evidence was restricted to the self-referential processing condition. In contrast, in the other-referential processing condition, there was no evidence whatsoever of such depression-linked reduced attention to positive information. This pattern of results is consistent with cognitive theories of depression postulating that a depressogenic self-schema, which lacks positive information concerning the self, may drive the patterns of selective information processing associated with elevated depression. It is plausible that inconsistencies across previous studies, in terms of whether a depression-linked reduced attention to positive information has been observed, may reflect unintended variation across studies in whether participants tended to engage in the self-referential processing of the presented emotional information.

In the present study, there was no evidence that the observed depression-linked reduction in attention to positive information was moderated by stimulus exposure duration. If one adopts the assumption that patterns of attentional selectivity observed at shorter stimulus exposure durations reflect biased attentional engagement with emotional information, and patterns of attentional selectivity observed at longer stimulus exposure durations reflect biased attentional disengagement from emotional information, then the fact that this depression-linked attentional bias was equally evident at both the shorter and longer stimulus exposure duration invites speculation depression-linked reduction in attention to positive information may equally be carried by reduced engagement with, and increased disengagement from, positive information. Future research could directly test this possibility by using newly developed variants of the dot-probe task specifically configured to dissociate these two facets of attentional selectivity (e.g. Grafton and MacLeod, 2014, Rudaizky et al., 2014).

In keeping with previously reported findings using the dot-probe task (e.g. Bradley et al., 1997, Shane and Peterson, 2007), and other tasks that assess attentional deployment (e.g. McCabe, Gotlib, & Martin, 2000), both the high and low depression groups in the present study showed a general tendency to direct attention away from negative stimuli. However, of importance to the issue under consideration, there was no evidence that depression-linked attentional responding to negative information was affected by whether this information had been processed in a self- or other-referential manner. Thus, there was no support for the idea that biased attentional responding to negative information in depressed individuals is driven by a depressogenic self-schema that is disproportionately negative in content. Interestingly, there was greater evidence of a depression-linked attentional bias to negative information at the longer stimuli exposure duration than at the shorter exposure duration, with stimulus exposure duration significantly moderating the impact of the depression group factor in this analysis. This suggests that depression-linked attentional bias to negative information may be more strongly characterized by impaired attentional disengagement from, rather than by enhanced attentional engagement with, negative information. This pattern of findings also lends weight to the argument that variation in stimulus exposure duration may account for some of the inconsistencies across past studies, in terms of whether a depression-linked attentional bias to negative information has been observed.

These present findings are the first to demonstrate that self vs. other referential processing impacts on depression-linked attentional responding to positive information. Social psychology research on self versus other judgments have shown that, while healthy individuals exhibit a robust positive “illusory glow” in beliefs and judgments about the self in comparison to others (Lewinsohn, Mischel, Chaplin, & Barton, 1980, c.f.; Mezulis, Abramson, Hyde, & Hankin, 2004), depressed individuals show comparatively reduced self-enhancement bias (Alloy & Ahrens, 1987), and a higher tendency to evaluate the self unfavorably in comparison to others (Butzer and Kuiper, 2006, Wood and Lockwood, 1999). Clearly, a pattern of attentional selectivity involving greater avoidance of positive information in general would not contribute to such a pattern of depression-linked self vs. other evaluations. However, greater attentional avoidance of positive information that has been processed in a self-referential manner, relative to positive information that has been processed in an other-referential manner, could indeed contribute to unfavorable comparisons between the self and others, as is characteristic of depressogenic thinking. Future research can now employ the experimental paradigm introduced in the present study to directly test whether depression-linked attentional avoidance of self-referential relative to other-referential positive information contributes to depression-linked individual differences in social comparative judgment.

In addition to advancing theoretical understanding of positive information processing in depression, our findings may have potential applied implications, particularly with respect to optimizing the design of newly emerging attentional bias modification (ABM) approaches to the alleviation of depression (c.f. MacLeod & Mathews, 2012). The ABM approach involves exposing participants to training versions of tasks such as the present dot-probe procedure, now configured to change attentional selectivity by consistently either presenting probes proximally to information that it is intended to train participants to attend towards, and/or presenting probes distally to information that it is intended to train participants to attentionally avoid. Studies that have attempted to alleviate depression by using ABM to induce increased attention to positive information in clinical samples have yielded mixed findings. While some such studies have elicited reductions in depression symptoms (Browning et al., 2011, Browning et al., 2012), others have failed to attenuate depression in this manner (Baert, De Raedt, Schacht, & Koster, 2010). The present findings suggest the possibility that the efficacy of ABM training, designed to ameliorate depression by changing attentional responding to positive information, might be enhanced by ensuring that recipients engage in the self-referential processing of this positive information during the ABM training procedure. Training that increases attention towards positive information overall, rather than towards self-referentially processed positive information, may yield no benefit in terms of depression reduction. Indeed, it is possible that, if such training were to increase attention towards positive aspects of other individuals, without reducing depression-linked attentional avoidance of self-referentially processed positive information, then this may exacerbate the maladaptive social comparisons known to characterize depressive vulnerability and dysfunction (Lyubomirsky and Ross, 1997, Swallow and Kuiper, 1988).

Of course, the present study has a number of limitations. First, the number of participants was relatively small, and so it will be important for future research to replicate the present study with larger participant samples. Second, the current results have been obtained using only a single measure of attentional deployment, the dot-probe task, and some researchers have raised concerns about the reliability of this task (Schmukle, 2005, Waechter et al., 2014). Thus, any future replications should seek to obtain converging evidence for our current conclusions, ideally by employing multiple methods of measuring attentional selectivity, and eye-tracking approaches offer particular promise. Thirdly, although not strictly necessary for testing the hypothesis under present scrutiny, the inclusion of a baseline condition that did not involve either self-referential or other-referential processing of the stimulus words could serve to strengthen future research intended to extend the present work. However, decisions concerning the appropriate baseline condition would need to be taken with care. For example, simply omitting any direct instructions concerning whether to engage in self-referential or other-referential processing in such a baseline condition would not suffice to preclude the possibility that such processing nevertheless may occur spontaneously. However, the prospect of referential processing taking place could be attenuated by requiring participants to engage in the structural, rather than the semantic processing, of stimulus words in such a baseline condition. While this approach may preclude self-referential and other-referential processing in this baseline condition, it would also reduce depth of processing, potentially introducing a depth of processing confound between the baseline and experimental conditions. Thus, decisions concerning the choice of a baseline condition should be guided by the specific nature of those future experimental questions that cannot be adequately resolved without including a particular baseline condition.

It remains to be seen whether the present findings will be replicated within clinically depressed populations and, if so, whether the introduction of a self-referential processing requirement enhances the degree to which ABM procedures designed to increase attention to positive information can therapeutically contribute to the reduction of such dysfunction. For the moment, however, the present study confirms that elevated depression is characterized by reduced attention to positive information, but for the first time shows that this effect is only evident when such information has been self-referentially processed. The finding that depression-linked attentional bias to positive information is moderated by referential processing focus advances understanding of depression-linked attentional processing in a manner that may contribute to the refinement of more effective intervention techniques, such as those designed to reduce depression by therapeutically targeting the underlying patterns of cognitive bias.

## Figures and Tables

**Table 1:** Attentional bias to negative words.

Means and standard deviations of attentional bias to negative word index scores (in ms)				
Group	Stimulus exposure duration			
	500 ms		1000 ms	
	M	SD	M	SD
High Depression				
Self-Referential	−32.84	48.13	−14.05	40.56
Other-Referential	−51.86	44.30	−18.96	22.78
Low Depression				
Self-Referential	2.11	57.08	−33.74	30.40
Other-Referential	−23.43	43.50	−31.57	46.94

**Table 2:** Attentional bias to positive words.

Means and standard deviations of attentional bias to positive word index scores (in ms)				
Group	Stimulus exposure duration			
	500 ms		1000 ms	
	M	SD	M	SD
High Depression				
Self-Referential	−44.59	36.86	−22.45	44.42
Other-Referential	2.07	40.40	−15.23	54.59
Low Depression				
Self-Referential	−4.71	90.31	9.37	20.88
Other-Referential	−40.31	32.24	−9.04	34.89

## Footnotes

1 In keeping with Koster, Crombez, Verschuere, and De Houwer (2004), across all conditions, RTs were excluded if they fell below 200 ms or above 2000 ms, and in keeping with Rudaizky et al. (2014), within each condition, RTs were excluded if they fell more than 2.58 SD away from the participant's mean RT for that condition.
